# Effects of *in vitro* simulated digestion on the α-glucosidase inhibitory activity, structure, and prebiotic activity of a polysaccharide from *Anemarrhena asphodeloides* Bunge

**DOI:** 10.3389/fnut.2025.1603237

**Published:** 2025-08-08

**Authors:** Baolian Li, Guihong Fang, Meijuan Lan, Juan Xiao, Xia Zhang, Lin Li, Bing Li, Juncheng Chen

**Affiliations:** ^1^Heinz Mehlhorn Academician Workstation, Department of Nutrition and Food Hygiene, School of Public Health, Hainan Medical University, Haikou, Hainan, China; ^2^School of Food Science and Engineering, Guangdong Province Key Laboratory for Green Processing of Natural Products and Product Safety, Engineering Research Center of Starch and Plant Protein Deep Processing, Ministry of Education, South China University of Technology, Guangzhou, China; ^3^Medical Devices Research & Testing Center of SCUT, South China University of Technology, Guangzhou, China

**Keywords:** *Anemarrhena asphodeloides* Bunge polysaccharide, α-glucosidase, digestive property, structure, intestinal microbiota

## Abstract

This study explored the changes in the structure and α-glucosidase inhibitory activity of a non-starch polysaccharide derived from *Anemarrhena asphodeloides* Bunge, AABP-1B, during digestion *in vitro* and its effect on host intestinal microbiota. Simulations of digestion in the upper digestive tract showed that the reducing sugar content and molecular weight of AABP-1B changed slightly, though no monosaccharides were detected. AABP-1B was resistant to degradation in the simulated upper gastrointestinal environments, retained strong α-glucosidase inhibitory activity after digestion, which may be related to the lack of structural changes. In *in vitro* fermentation, AABP-1B enhanced the growth of commensal microorganisms, including *Bacteroides*, *Megasphaera*, and *Prevotella*, while inhibiting the proliferation of pathogenic bacteria, such as *Escherichia-Shigella*. Fermentation of AABP-1B by gut microbes resulted in a notable increase in short-chain fatty acid contents and a decrease in pH levels. Our findings showed that AABP-1B promotes intestinal health and may serve as a prebiotic in the development of functional food.

## 1 Introduction

Human health and the gut microbiome are shaped by factors such as diet, gastrointestinal conditions, and genetics, which influence bacterial composition. Genome-wide association studies have linked genetic loci, including LCT (lactase persistence) and FUT2 (secretor status), to the abundance of specific microbial taxa (1, 2). Alterations in intestinal microbiota composition have been associated with the development of type 2 diabetes mellitus (3, 4). Diabetes, primarily type 2 (95% of cases), is a chronic disease linked to abnormal insulin function and poses a significant global health threat. Normal intestinal flora helps maintain the integrity of the intestinal mucosa (5). However, in diabetic patients, the imbalance of intestinal flora may lead to impaired intestinal barrier function, allowing harmful substances such as endotoxins in the intestine to enter the blood circulation, triggering chronic inflammatory responses. This chronic inflammation will interfere with insulin signaling, reduce the effect of insulin, and further aggravate the condition of diabetes (6, 7). In addition, intestinal flora can produce metabolites such as short-chain fatty acids, which can participate in the regulation of host glucose metabolism. For example, butyric acid can promote the uptake and utilization of glucose by intestinal cells, while the excessive growth of certain harmful bacteria may reduce the production of short-chain fatty acids, affecting the normal progress of sugar metabolism. Dietary polysaccharides may be used as natural bioactive prebiotics for enhancing intestinal health (8, 9). Regulating intestinal microbiota, and inhibiting glucose metabolism-related enzyme (such as α-glucosidase and α-amylase) activities, thereby improving blood glucose regulation and ameliorating diabetic symptoms (10, 11). Floris et al. (12) suggested that inhibiting α-glucosidase activity reduced the breakdown of dietary carbohydrates into monosaccharides, aiding in the management of postprandial blood glucose surges. The physicochemical properties of some non-starchy carbohydrates are influenced by bile salts, digestive enzymes, and pH during digestion *in vitro* (13). Nonetheless, the breakdown of polysaccharides into oligosaccharides or monosaccharides in the human upper gastrointestinal tract is limited by the low levels of carbohydrate-active enzymes (CAZymes). Subsequently, polysaccharides come into contact with a wider range of CAZymes in the colon and are metabolized by gut microbiota to generate short-chain fatty acids (SCFAs) (14, 15), which provide various health benefits (16, 17). These acids mitigate inflammation, and diabetes while also influencing brain function (18–20). Polysaccharides from *Grifola frondosa* (21) and other mushrooms (22) are resistant to digestion but can be broken down through intestinal fermentation, producing SCFAs along with other metabolites. These polysaccharides promote the proliferation of beneficial bacteria and impede the growth of spoilage bacteria, thereby modulating gut microbiota composition. The biological activity of polysaccharides has been widely studied in animal models, demonstrating their potential to influence gut health, immune function, and metabolism (23–27). FTZPs exert protective effects by modulating the gut microbiota, reducing the abundance of *Gammaproteobacteria*, and increasing the abundance of *Dehalobacteraceae* and *Dehalobacterium*, while also restoring intestinal barrier function (28). Additionally, FTZPs enhance the level of asparagine, further contributing to their protective effects. Similarly, *Stevia rebaudiana root* polysaccharides promote the growth of beneficial bacteria such as *Lactobacillus* and *Bifidobacterium*, modulating gut health and improving liver metabolism, thereby alleviating the symptoms of non-alcoholic fatty liver disease (NAFLD) (29). Given their various benefits and roles in human physiology, it is important to characterize the prebiotic activity of polysaccharides.

*Anemarrhena asphodeloides* Bunge (AAB) is a perennial herb of the genus *Asphodeloides*, which has antitumor, antiviral, antimicrobial, antioxidant, anti-inflammatory, anti-osteoporosis, anti-skin-aging, and cytoprotective properties. AAB-derived polysaccharides have been reported to protect nerve function and reduce blood glucose levels. We previously isolated a non-starch polysaccharide (AABP-1B, 105 kDa) from AAB that contained 4)-2-*O*-acetyl-β-d-Man*p*-(1 and 4)-β-d-Man*p*-(1 glycosidic bonds and showed strong inhibitory activity against α-glucosidase. Structural analysis of AABP-1B suggested that it may resist degradation in the upper gastrointestinal tract, potentially reaching the colon for metabolism by gut microbiota (30). However, the digestion and transformation of polysaccharides are complex, and it remains unclear whether AABP-1B retains its α-glucosidase inhibitory activity post-digestion and whether its fermentation by intestinal microbiota benefits the host.

The purpose of this study was to investigate the effects of simulated upper gastrointestinal digestion on the structural properties and α-glucosidase inhibitory activity of AABP-1B. Subsequently, the effects of AABP-1B on intestinal microbiota and SCFAs were studied by simulating *in vitro* fermentation models. Our findings provide valuable insights into the potential application of AABP-1B as a prebiotic in functional foods for modulating the gut microbiota and a useful reference for developing clinical strategies against diabetes.

## 2 Materials and methods

### 2.1 Materials and reagents

The AAB was purchased from a local pharmacy in Guangzhou (Guangdong, China). Calcium chloride, chloroform, sodium hydroxide, anhydrous ethanol, trifluoroacetic acid, and methanol were purchased from Xilong Scientific (Guangdong, China). α-glucosidase (100 U/mg) and short-chain fatty acid standards were sourced from Sigma-Aldrich (St. Louis, MO, United States). Other chemical reagents used were of analytical grade.

### 2.2 Extraction and purification

The extraction and isolation of AABP was performed as previously described (30). The dried AAB powder was first reflux-extracted with 95% ethanol at 80°C for 4 h to remove pigments and lipids. After filtration and drying, the residue was extracted with distilled water (1:25 w/v) at 90°C for 3 h. This hot-water extraction was repeated three times, and the combined aqueous extracts were collected. The water-based extract was then centrifuged, and the supernatant was concentrated for further processing. Anhydrous ethanol was added dropwise to the supernatant up to a concentration of 60% and left for 24 h at 4°C before centrifugation. The precipitate was deproteinized using the Sevag method and subsequently dialyzed and freeze-dried to obtain crude polysaccharide (AABP). AABP was dissolved in distilled water and filtered using a 5-μm membrane. The filtrate was purified using a DEAE-52 column (2.6 × 40.0 cm) and eluted with 0, 0.1, 0.2, 0.4, and 0.5 mol/L NaCl at a flow rate of 1.0 mL/min. The 0.2-mol/L NaCl elution peaks were collected and subjected to dialysis and lyophilization to generate AABP-1B.

### 2.3 *In vitro* simulation of AABP-1B digestion

#### 2.3.1 Simulated salivary digestion of AABP-1B

Artificial saliva was prepared following the previously described method, with appropriate modifications (31). Artificial saliva was formulated by dissolving 0.7644 g/L NaCl, 1.491 g/L KCl, and 0.1332 g/L CaCl₂ in 1 L distilled water. The pH was precisely adjusted to 6.9 ± 0.05 using 1 M HCl. The oral digestive solution was formulated by supplementing 150 mL artificial saliva with 1% (w/v) α-amylase. We combined 30 mL AABP-1B (20.0 mg/mL) with 30 mL artificial saliva for incubation (37°C, 100 rpm). Samples (2.0 mL) were extracted at 0 h and 0.5 h, subsequently subjected to enzyme inactivation by boiling for 5 min, and the remaining liquid was reserved for gastric digestion simulation.

#### 2.3.2 Gastric digestion simulation of AABP-1B

Artificial gastric fluid was prepared following an established protocol (32). The electrolyte base (pH 2.0 ± 0.1) contained 1.1 g/L KCl, 3.1 g/L NaCl, 0.15 g/L CaCl₂, and 0.6 g/L NaHCO₃. Gastric digestive fluid was subsequently generated by integrating lipase (0.19 mg/mL), pepsin (0.18 mg/mL), and sodium acetate (0.75% v/v) into the electrolyte matrix. Saliva-digested samples were mixed with the gastric digestive fluid in equal volumes and shaken (37°C, 100 rpm). Gastric digestive samples (2.0 mL) were obtained at 2, 4, and 6 h and subsequently subjected to enzyme inactivation by boiling for 5 min. After 6 h of gastric digestion, the pH of the digestive juice was adjusted to 7.5 using NaOH solution (0.2 M).

#### 2.3.3 Simulated intestinal digestion

Artificial small intestinal fluid was prepared following an established protocol with appropriate modifications (32). The simulated intestinal electrolyte was formulated by dissolving 0.65 g/L KCl, 5.4 g/L NaCl, and 0.33 g/L CaCl₂ in 1 L distilled water, with pH adjusted to 7.0 ± 0.1 using 1 M NaOH. Digestive fluid was prepared by supplementing 250 mL electrolyte with pancreatic enzyme (0.4 mg/mL) and 4% bile solution (31.25 mL, 12.5% v/v). Gastric-digested samples were mixed with intestinal digestion solution at equal volumes and incubated in a shaker (37°C, 100 rpm). Samples (2.0 mL) were collected at 2, and 4 h and heat-treated in boiling water for 5 min to deactivate the enzymes. We determined the reducing sugar, dissociated monosaccharide contents, and molecular weight of inactivated digestive samples collected from the saliva, stomach, and intestine.

### 2.4 Molecular weight (mw), reducing sugar (C_R_) and monosaccharide composition during AABP-1B digestion

High-performance gel permeation chromatography (HP-GPC) was employed to ascertain the *Mw* of AABP-1B during digestion. Digestive fluid samples were filtered before analysis. The molecular weight was determined by HP-GPC using different dextran standards (13.05, 36.80, 64.65, 135.35, 300.60, and 670.00 KDa) as the calibration reference. The chromatographic column and detection conditions adhered to those previously described (32). The reducing sugar content test is performed according to the method provided in the reducing sugar test kit (BC0230, Solarbio, Beijing, China). The monosaccharide composition of the digested AABP-1B samples was analyzed using high-performance liquid chromatography (HPLC). Both the standard monosaccharides and the digested AABP-1B solution were derivatized with 1-phenyl-3-methyl-5-pyrazolone (PMP). The PMP-derivatized samples were chromatographically resolved using a Waters Symmetry C18 column (5 μm particle size, 4.6 × 250 mm) maintained at 30°C (30). The chromatogram was recorded using a Waters 2,998 system (Waters, United States) equipped with a 2,489 UV–vis detector at 245 nm. The mobile phase consisted of 0.05 mol L^−1^ KH_2_PO_4_ (0.05 M, pH 6.8) and acetonitrile (83: 17, v/v), delivered at a flow rate of 1.0 mL/min. The column temperature was maintained at 30°C, and the injection volume was set to 20 μL.

### 2.5 Inhibition of α-glucosidase activity by AABP-1B before and after digestion

The inhibitory activity of AA on α-glucosidase was assessed before digestion and after simulated intestinal digestion, as previously described (33). AABP-1 solutions were mixed with 100 μL α-glucosidase solution (0.8 U/mL), incubated at 37°C for 10 min, treated with 100 μL p-Nitrophenyl-α-d-glucopyranoside (pNPG, 10 mM), and reacted for 30 min at 37°C. The reaction was terminated using 100 μL Na_2_CO_3_ (1 M). The absorbance of the reaction solution was detected at 405 nm using a microplate reader. The α-glucosidase inhibition rate (%) was calculated using the following formula:

Inhibition rate (%) = [1 − (Es − Ec)/Eb] × 100.

where Es is the absorbance of the reaction solution containing polysaccharide sample, pNPG (substrate), and α-glucosidase; Ec is the absorbance of the reaction solution in which phosphate buffer replaces the polysaccharide sample; Eb is the absorbance of the solution in which phosphate buffer replaces α-glucosidase.

### 2.6 Simulated *in vitro* fermentation

The *in vitro* fermentation of AABP-1B was simulated as previously described (34). Fresh fecal samples were collected from five healthy, asymptomatic adults (three males and two females, aged 25–40 years) who had not taken antibiotics or probiotics for at least 3 months. A 10% fecal slurry. The fermentation medium was prepared by dissolving the following components in 1 L distilled water: 10 mg/L MgSO₄·7H₂O, 2.0 g/L yeast extract, 0.1 g/L NaCl, 40 mg/L KH₂PO₄, 10 mg/L CaCl₂, 40 mg/L K₂HPO₄, 2.0 g/L peptone, 2.0 g/L NaHCO₃, 20 mg/L heme, 0.5 g/L cysteine hydrochloride, 0.5 g/L bile salt, 2.0 mL/L Tween 80, 1.0 mL/L 1% (w/v) resazurin solution, and 10 μL/L vitamin K. The culture medium was treated with 0.1 mol/L HCl solution to a pH of 7.0 and sterilized at 121°C for 20 min. In the carbon-source group, 2 mL of fecal suspension was added to 18 mL fermentation medium containing 10 mg AABP-1B. Inulin (IN) and sterile water were utilized as positive and blank (BLK) controls, respectively. The treated samples were cultured in an anaerobic incubator at 37°C. Samples were taken at 0, 6, 12, and 24 h to assess the SCFA content, pH, and microbial composition.

### 2.7 pH and SCFA content analyses

The pH of the fermentation supernatant was determined using an acidity meter (PHSJ-3F, Leici, Shanghai, China). To extract SCFAs, 1.0 mL of the fermentation broth was mixed with 1.0 mL of distilled water, followed by the addition of 1.0 mL of diethyl ether for extraction. The extraction was conducted twice and the ethyl ether phase was combined and concentrated to 1.0 mL for gas chromatography–mass spectrometry (GC–MS) performed with the following conditions: carrier gas (nitrogen) flow rate, 19 mL/min; initial column temperature, 100°C for 1 min, increased at 4°C/min to 180°C for 4 min; detector and inlet temperatures, 250°C; electron beam energy, 70 eV, ion source temperature, 200°C, *m/z* range, 40–800.

### 2.8 Gut microbiota analysis

The total DNA of bacteria in the fermentation samples was extracted using a E. Z. N. A. Stool DNA Kit (Omega Bio-Tek, Jiangsu, China) according to the manufacturer’s protocols. An appropriate sample quantity was placed in a centrifugation tube for PCR amplification of the V3–V4 variable sequence area using 806R and 338F primers. Gel electrophoresis was conducted with fluorescently labeled nucleic acids, and target bands were selected in accordance with the cartridge instructions. DNA purification and recovery from gels were performed using DNA Gel recovery kits, including a QIAquick Gel Extraction Kit (QIAGEN, Hilden, Germany). DNA concentration was determined using a fluorometer. MiSeq high-throughput sequencing (Illumina, San Diego, CA, United States) was employed for quality control and sequencing analysis. The α- and β-diversity indices were estimated using Quantitative Insights into Microbial Ecology based on the sequencing reads and operational taxonomic units (OTUs).

### 2.9 Statistical analysis

All experiments were repeated three times and the data are expressed as the mean ± standard deviation. Statistical analysis and graphical representation of the data were performed using SPSS 27 and OriginPro 2019 software.

## 3 Results

### 3.1 Changes in Mw, free monosaccharide, and reducing sugar (CR) concentrations of AABP-1B during digestion

**Figure 1:**
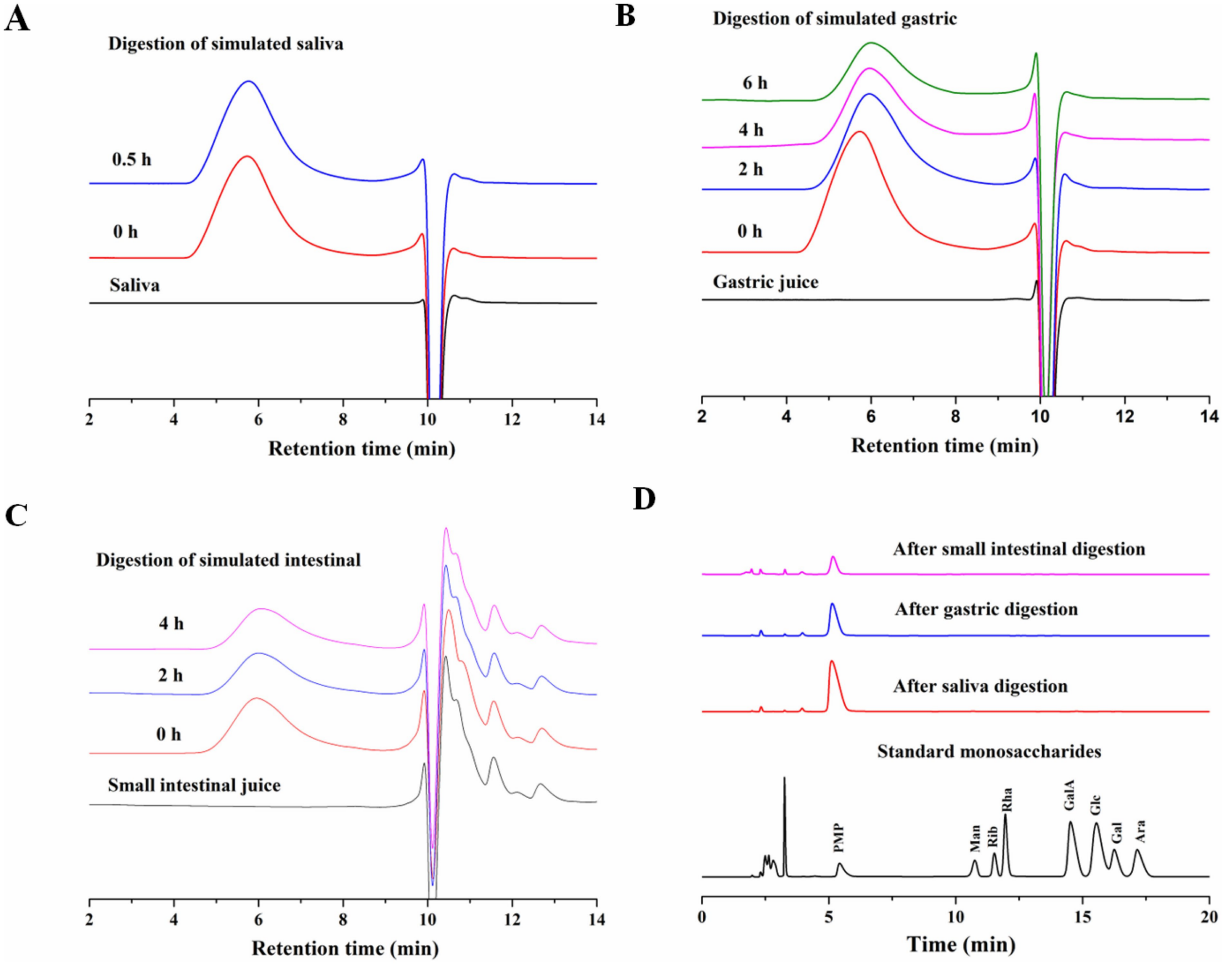
**(A)** Changes in Mw of AABP-1B during simulated digestion in saliva; **(B)** stomach; **(C)** and intestinal fluid; **(D)** changes in free monosaccharide contents during digestion.

The oral cavity serves as the initial site of food digestion, where salivary amylase enzymatically cleaves α-1 → 4 glucoside bonds in starch and other carbohydrates. However, non-starch polysaccharides are not susceptible to hydrolysis by salivary amylases. The release of free monosaccharides from AABP-1B during gastrointestinal digestion is presented in Figure 1D. However, comparative analysis with standard monosaccharide profiles revealed that no free monosaccharides were detected throughout the entire digestion process. Non-starch polysaccharides from snow chrysanthemum (31) and wolfberry (34) are resistant to degradation by human saliva, likely due to the absence of α-1 → 4 glucoside bonds limiting their hydrolyzation by α-amylase. Our findings suggested that the stability of the 4)-2-O-acetyl-β-d-Man*p*-(1 and 4)-β-d-Man*p*-(1 glycosidic bonds is not affected by salivary amylase (30).

**Figure 2:**
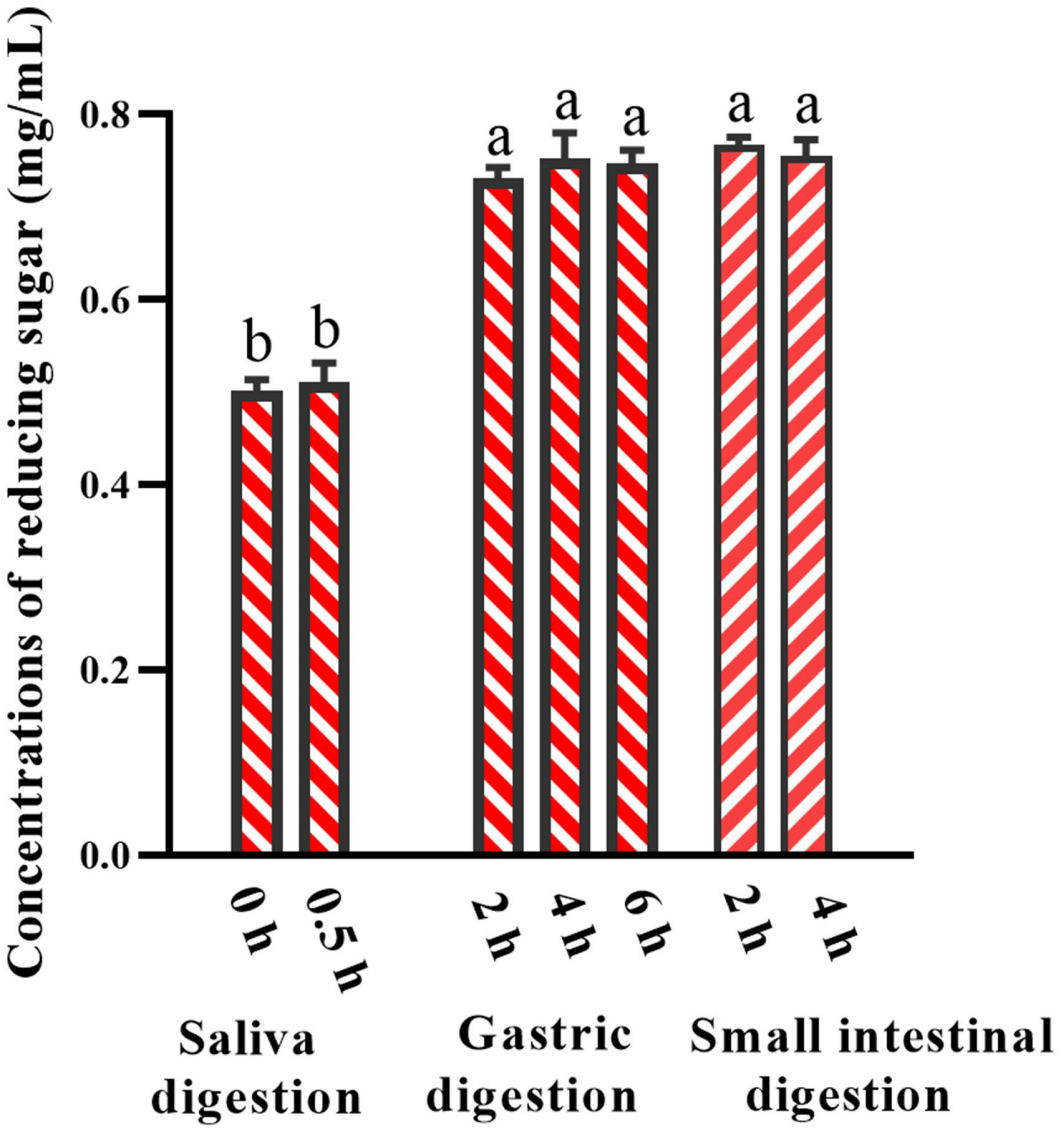
Changes in reducing sugar contents in simulated digestion. Values were mean ± SD (*n* = 3). Different superscript letters within columns indicate differences (*p* < 0.05).

Most polysaccharides are resistant to breakdown in the upper gastrointestinal tract. The *Mw* changes of AABP-1B during oral digestion is shown in Figures 1A–C. Following 0.5 h of simulated salivary digestion, the *Mw* of AABP-1B did not change significantly and the C_R_ concentration remained constant (Figures 1A, 2). After 2 h of simulated gastric digestion, the *Mw* of AABP-1B declined significantly from 105.8 ± 0.43 KDa to 98.2 ± 0.24 KDa (Figure 1B) while the C_R_ concentration increased from 0.511 ± 0.021 mmol/L to 0.732 ± 0.011 mmol/L (Figures 1B, 2). Following the simulated intestinal digestion, no significant changes were observed in the molecular weight or concentration of C_R_, and no free monosaccharides were detected (Figures 1C, 2). Detailed data are provided in Appendix Table 1 for reference. Collectively, these results demonstrate that simulated gastric digestion induced slight degradation of AABP-1B, leading to a marginal reduction in its molecular weight. The gastrointestinal digestibility of natural polysaccharides may vary depending on the source. The structure of some polysaccharide changes during *in vitro* simulated digestion in the stomach and small intestine, thereby decreasing the molecular weight of polysaccharides and increasing the contents of reducing sugars (35, 36). During digestion of food, intestinal fecal microbiota consumes carbohydrates to varying degrees, leading to the breakage of glycosidic bonds in polysaccharides and exposure of reducing end-groups, resulting in the production of several reducing sugars, which are then used as a carbon source for the growth of intestinal microbiota. Collectively, AABP-1B showed obvious resistance to both gastric and small intestinal digestion, and its structural integrity remained basically unchanged during transportation through the upper gastrointestinal tract. During subsequent fermentation phases, thereby potentiating its bioavailability for gut microbiota-mediated metabolic utilization.

### 3.2 Inhibitory effect of AABP-1B on α-glucosidase activity after digestion

**Figure 3:**
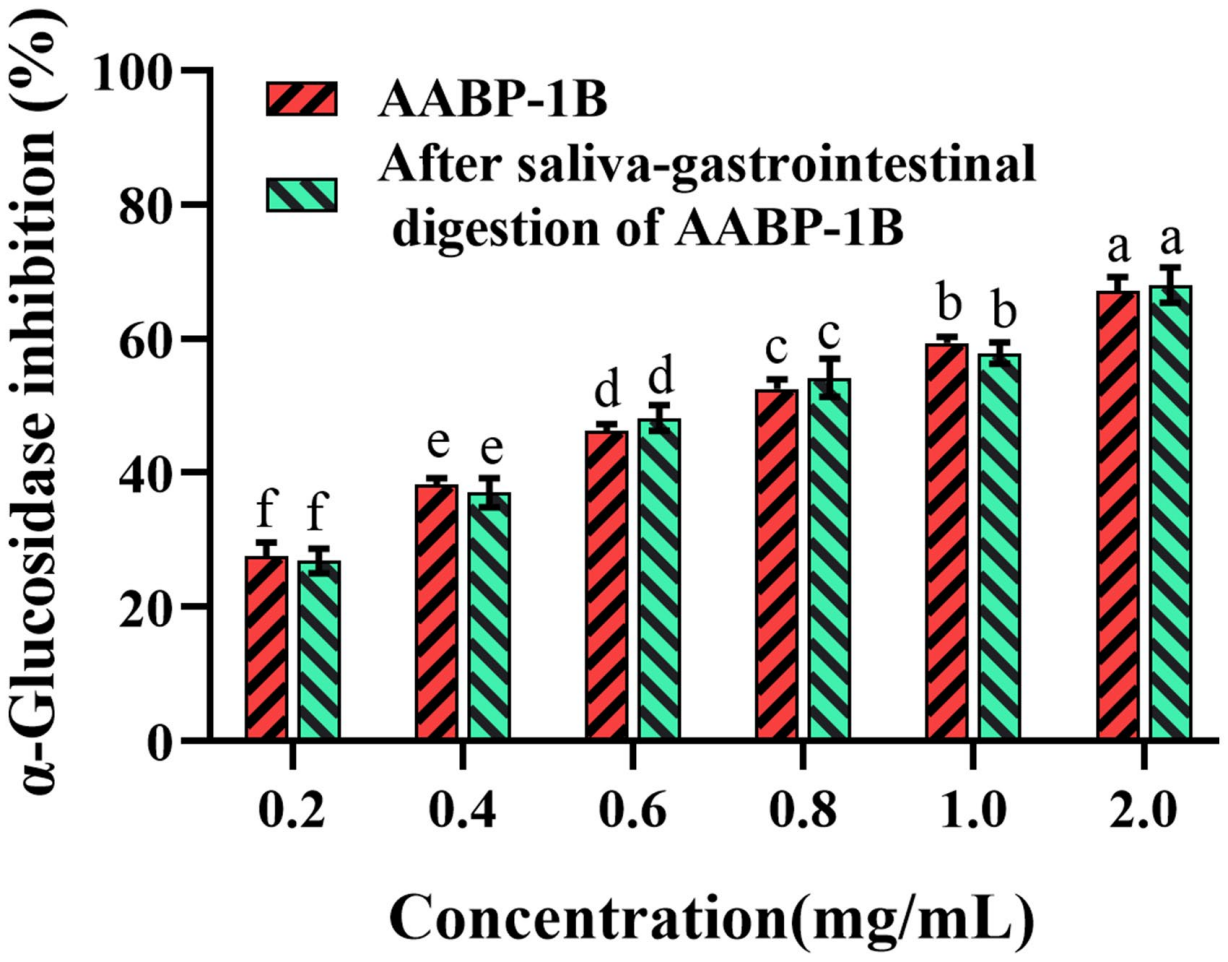
Inhibitory activity of AABP-1B on α-glucosidase before and after digestion; Values represent mean ± standard deviation. Different superscript lowercase letters indicated significance (*p* < 0.05) in each column.

The inhibition of α-glucosidase can attenuate postprandial high blood sugar levels by delaying the release of glucose in the small intestine. AABP-1B exhibited significant α-glucosidase inhibitory activity both before and after simulated digestion, showing a clear dose-dependent effect with no significant differences observed between pre- and post-digestion activity levels (Figure 3). Non-linear curve fitting revealed an IC₅₀ value of 61.5% ± 0.0527, with no significant difference before and after digestion. The stability of its inhibitory activity of AABP-1B may also be associated with specific glycosidic linkages and molecular conformations that safeguard the active sites of AABP-1B against enzymatic hydrolysis, thereby pre-serving its functional integrity. Hence, AABP-1B can potentially be used as a therapeutic agent in the treatment of diabetes or impaired glucose metabolism.

### 3.3 AABP-1B affects pH in fecal fermentation

**Figure 4:**
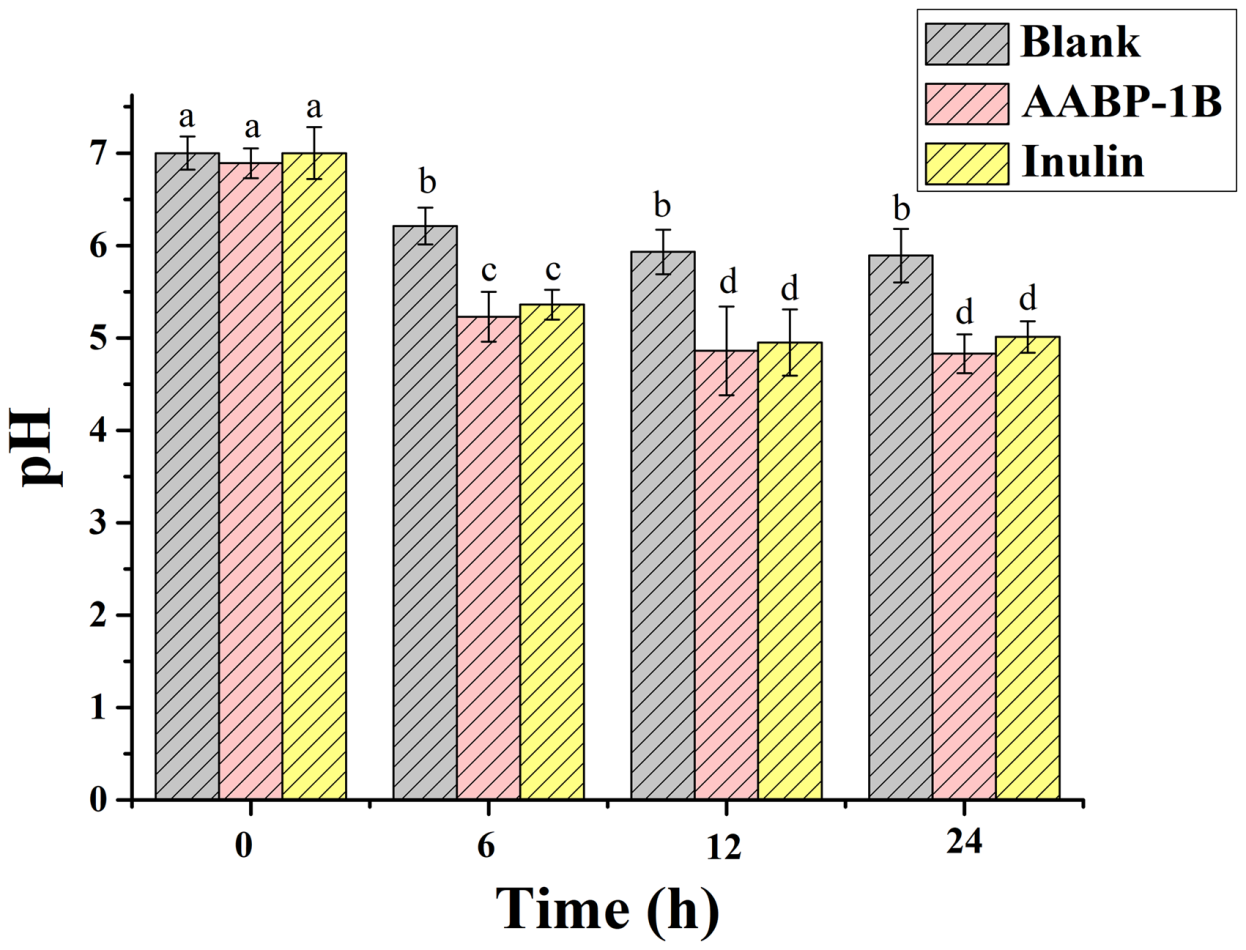
Variations in pH during fermentation of AABP-1B; values represent mean ± standard deviation; different superscript lowercase letters indicated significance (*p* < 0.05) in each column.

Polysaccharides that remain undigested by gastric or intestinal fluids may traverse the colon and serve as carbon substrates for intestinal microbiota, leading to the production of SCFAs, which play a crucial role in regulating intestinal pH and physiological homeostasis. As illustrated in Figure 4, the initial pH values of the AABP-1B group, inulin control group (IN), and blank control group (BLK) showed no statistically significant differences (*P* > 0.05). During the fermentation process, both the AABP-1B group and inulin groups demonstrated a significant pH decrease over the first 12 h (*p* < 0.05), followed by stabilization between 12 h and 24 h. Specifically, the pH values of the polysaccharide and inulin groups experienced a sharp decline within the first 6 h, eventually plateauing at approximately 4.86 and 4.95, respectively, by the 12 h. This finding is consistent with those of previous studies on the effects of *Pleurotus eryngii*-derived polysaccharides (37). The observed decrease in pH in the AABP-1B and inulin-treated groups may be attributed to their conversion to SCFAs. Lower pH levels may influence bacterial composition by promoting and suppressing the growth of beneficial and pathogenic bacteria, respectively (38).

### 3.4 Impact of AABP-1B on SCFA production in fecal fermentation

**Figure 5:**
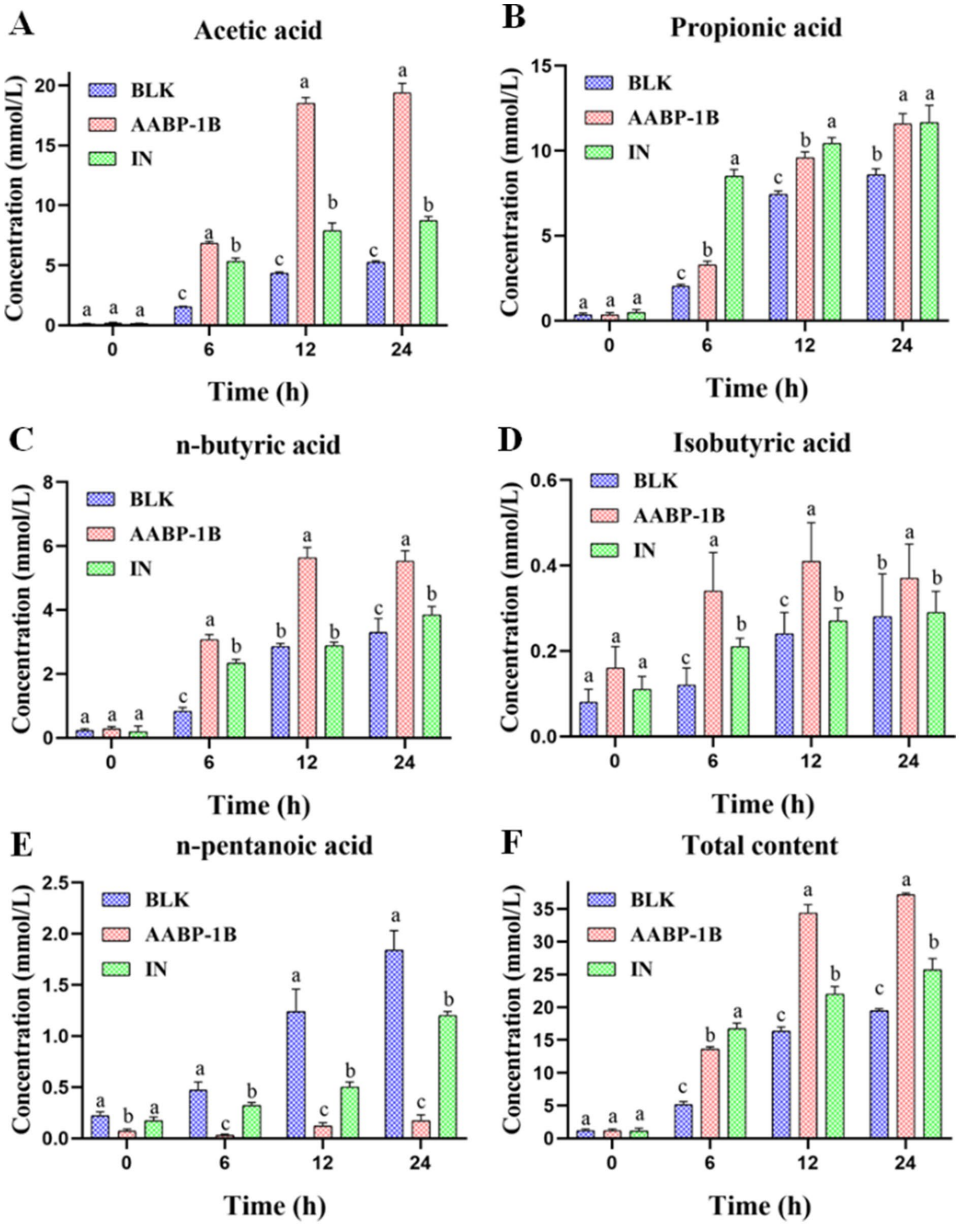
Variations in SCFA concentrations during fecal fermentation; values were mean ± SD (*n* = 3); different superscript letters within columns indicate differences (*p* < 0.05).

As shown in Figure 5F, in the BLK group, the total concentration of SCFAs increased from 1.18 ± 0.24 (0 h) to 19.48 ± 0.30 mmol/L (24 h). In the AABP-1B group, total SCFAs concentration increased significantly (*p* < 0.05), from 1.14 ± 0.27 mmol/L (0 h) to 37.15 ± 0.27 mmol/L (24 h). SCFAs produced by the fermentation of polysaccharides by intestinal microbiota are essential for maintaining intestinal health and regulating systemic metabolism. These effects extend beyond nutrient provision, and include immune regulation, antibacterial properties, and metabolic control (39, 40). SCFAs content in the fermentation solution of the AABP-1B and inulin groups were significantly (*p* < 0.05) higher than that in the BLK group at each time point, reflecting the effective production of SCFAs by intestinal microbiota through the fermentation of carbohydrates (Figure 5). Acetic, propionic, and butyric acids were identified as the primary metabolites in the AABP-1B group. Intestinal microorganisms can produce acetic, propionic, and n-butyric acid using hexose and pentose as energy substrates (41, 42). Additionally, propionic acid can be derived from deoxyhexose sugars such as rhamnose. Mannose (Man), Rhamnose (Rha), Galacturonic acid (GalA), Glucose (Glc), Galactose (Gal), and Arabinose (Ara). Components of AABP-1B serve as the primary substrates for SCFA synthesis. After fermentation for 24 h, acetic acid, propionic acid and n-butyric acid concentrations in AABP-1B group were significantly increased (*p* < 0.05) compared with the control group, which were 3.69-, 1.35-, and 1.67- times, respectively. Detailed data are provided in Appendix Table 2 for reference. Acetic acid regulates intestinal homeostasis, inhibits the invasion of harmful and opportunistic pathogenic microorganisms, and promotes the abundance and diversity of butyrate-producing bacteria (43). Butyrate maintains intestinal epithelial cell integrity, whereas propionate has been shown to reduce liver and plasma fatty acid levels, potentially enhancing tissue insulin sensitivity (43–45). Overall, AABP-1B exerted a prebiotic effect by promoting the synthesis of acetic, propionic, and n-butyric acids by gut microbiota.

### 3.5 Effects of AABP-1B on microbiome composition

**Figure 6:**
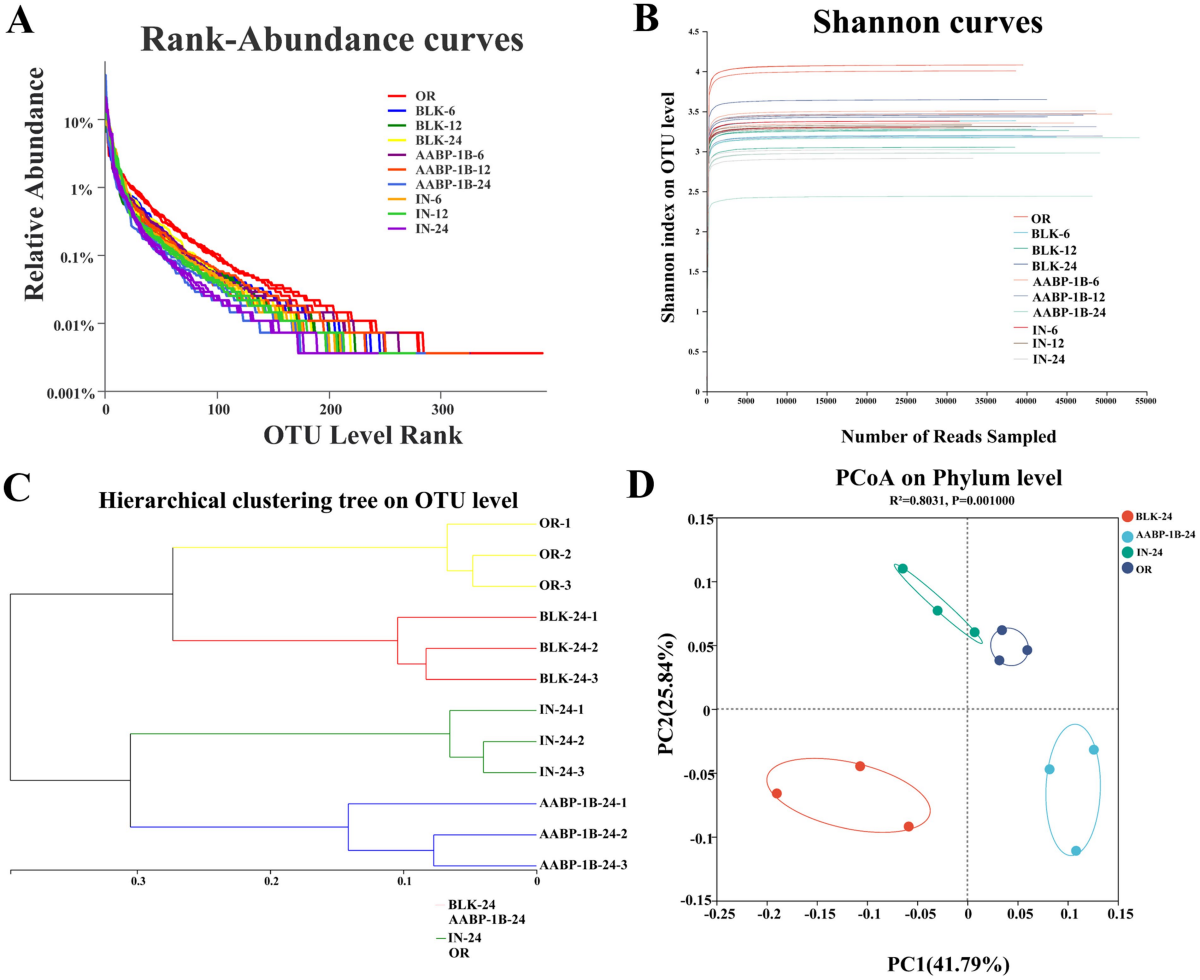
**(A)** Rank abundance; **(B)** Shannon curves; **(C)** hierarchical clustering tree based on OTUs; **(D)** PCoA.

Maintaining a balanced microbiome is essential for host health, particularly in terms of energy regulation, gut metabolism, and immune function. During anaerobic fermentation, polysaccharides act as carbon sources for gut microbiota growth, stimulate the growth of beneficial microbial populations, and facilitate the production of metabolites that enhance host health (14, 46). The effects of AABP-1B on the diversity, composition, and function of gut microbiota were investigated using high-throughput 16S rRNA sequencing. The rank-abundance curves (Figure 6A) and Shannon curves (Figure 6B) collectively demonstrated sufficient sequencing depth and effective coverage of microbial diversity (>99.88%). The rank-abundance curves showed a gradual flattening, particularly in the AABP-1B group at 24 h, indicating improved richness and evenness. Concurrently, the Shannon diversity index exhibited a decreasing trend from 6 to 24 h, suggesting a transition toward structural stabilization and the selective enrichment of microbial taxa capable of efficiently utilizing AABP-1B. The Bray–Curtis method indicated a marked divergence in the intestinal microbiota of the BLK and AABP-1B groups (Figure 6C), consistent with the principal coordinate analysis (PCoA) (Figure 6D), which demonstrated distinct clustering of intestinal microbiota among the experimental groups.

**Figure 7:**
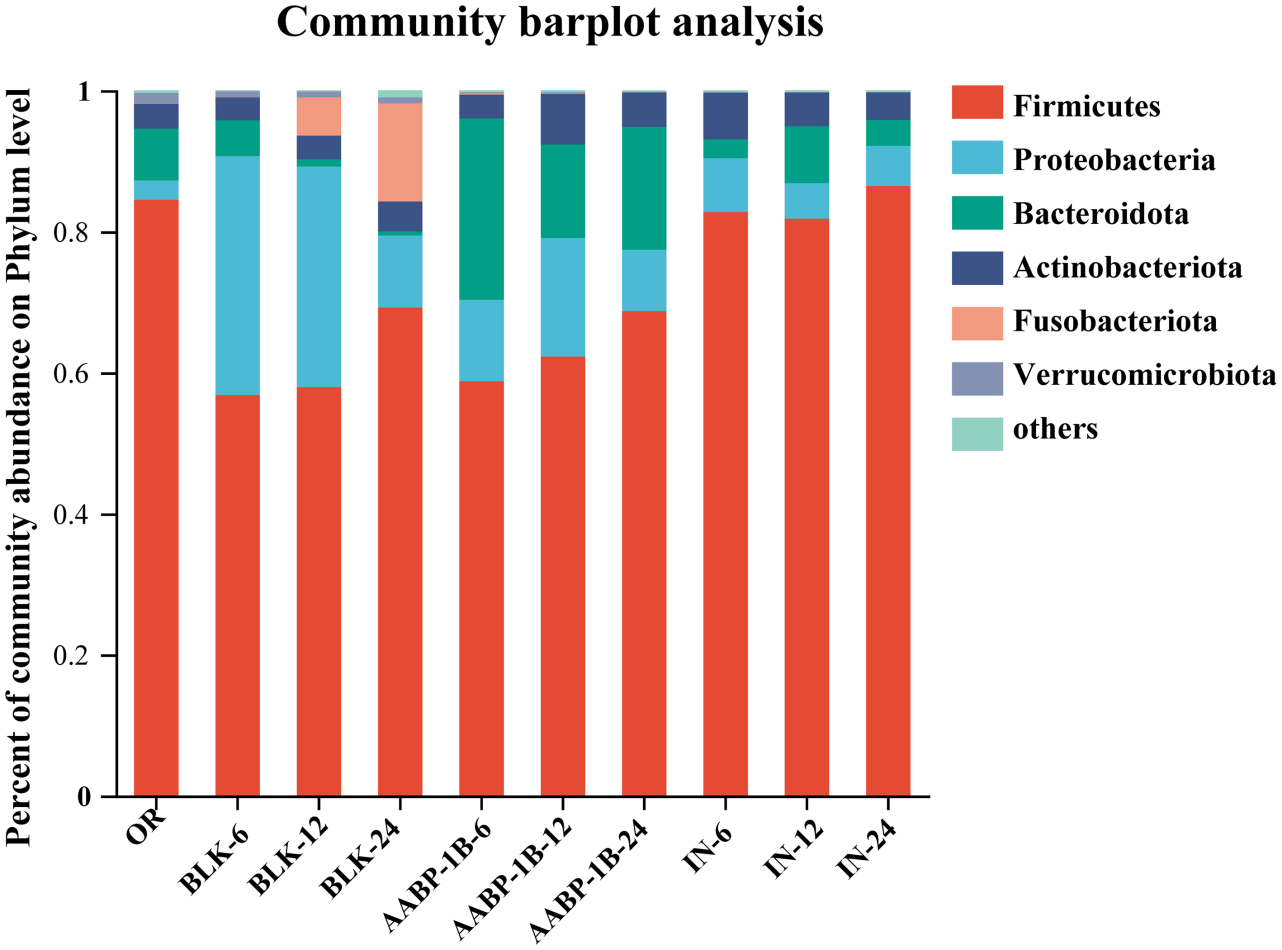
Phylum-level distribution of gut microbiota. OR was blank medium fermentation for 0 h; BLK-6, BLK-12, BLK-24, IN-6, IN-12, IN-24, AABP-1B-6, AABP-1B-12 and AABP-1B-24 were blank medium, Inulin medium, AABP-1B medium fermentation for 6 h, 12 h and 24 h, respectively.

The classification of the microbial composition at the phylum levels changed at 0, 6, 12, and 24 h of fermentation (Figure 7). The primary phyla detected among the gut microbiota were *Bacteroidetes*, *Firmicutes*, *Proteobacteria*, and *Actinobacteria* (Figure 7), consistent with prior findings (47). *Proteobacteria* is the most diverse bacterial phylum, encompassing both anaerobic and aerobic bacteria (48). The richness of *Proteobacteria* in the BLK, AABP-1B, and IN groups increased and decreased significantly after 6 and 24 h of fermentation, respectively. This may be due to the presence of trace amounts of oxygen during the initial stages of the fermentation process, despite stringent control of anaerobic conditions throughout the experiment, which promoted the proliferation of aerobic *Proteobacteria*. As fermentation time increased, trace oxygen was depleted, and *Proteobacteria* abundance in each experimental group decreased significantly. These results indicated that the fermentation process maintained robust anaerobic conditions. *Proteobacteria* comprise various pathogenic bacteria, including *Escherichia coli*, *Shigella*, *Salmonella*, and *Campylobacter*, which may disrupt intestinal microbiota balance, induce inflammation, and lead to chronic colitis (49, 50). Consequently, a lower abundance of *Proteobacteria* may be advantageous. *Proteobacteria* abundance was significantly lower in the AABP-1B or IN group after 24 h of fermentation compared with that in the BLK group. This suggests that fermentation of AABP-1B and IN can inhibit the proliferation of *Proteobacteria*. Certain *Bacteroidetes* and *Actinobacteria* can degrade and utilize polysaccharides to produce metabolites that promote the growth and proliferation of intestinal microbiota and maintenance of intestinal homeostasis (51). The relative abundance of *Bacteroidetes* was higher in the AABP-1B and IN groups than that in the BLK group at 24 h post-fermentation (*p* < 0.05). Body fat content is strongly associated with the *Firmicutes* to *Bacteroidetes* ratio, with lean individuals exhibiting a markedly lower ratio than obese individuals (52, 53). After 24 h of fermentation, the *Firmicutes*–*Bacteroidetes* ratio in the AABP-1B group was significantly lower than that in the BLK group (*p* < 0.05). These findings underscore the potential of AABP-1B in mitigating obesity and the associated intestinal inflammation. AABP-1B can also modulate gut microbiota composition by promoting beneficial bacterial growth while suppressing pathogenic species.

**Figure 8:**
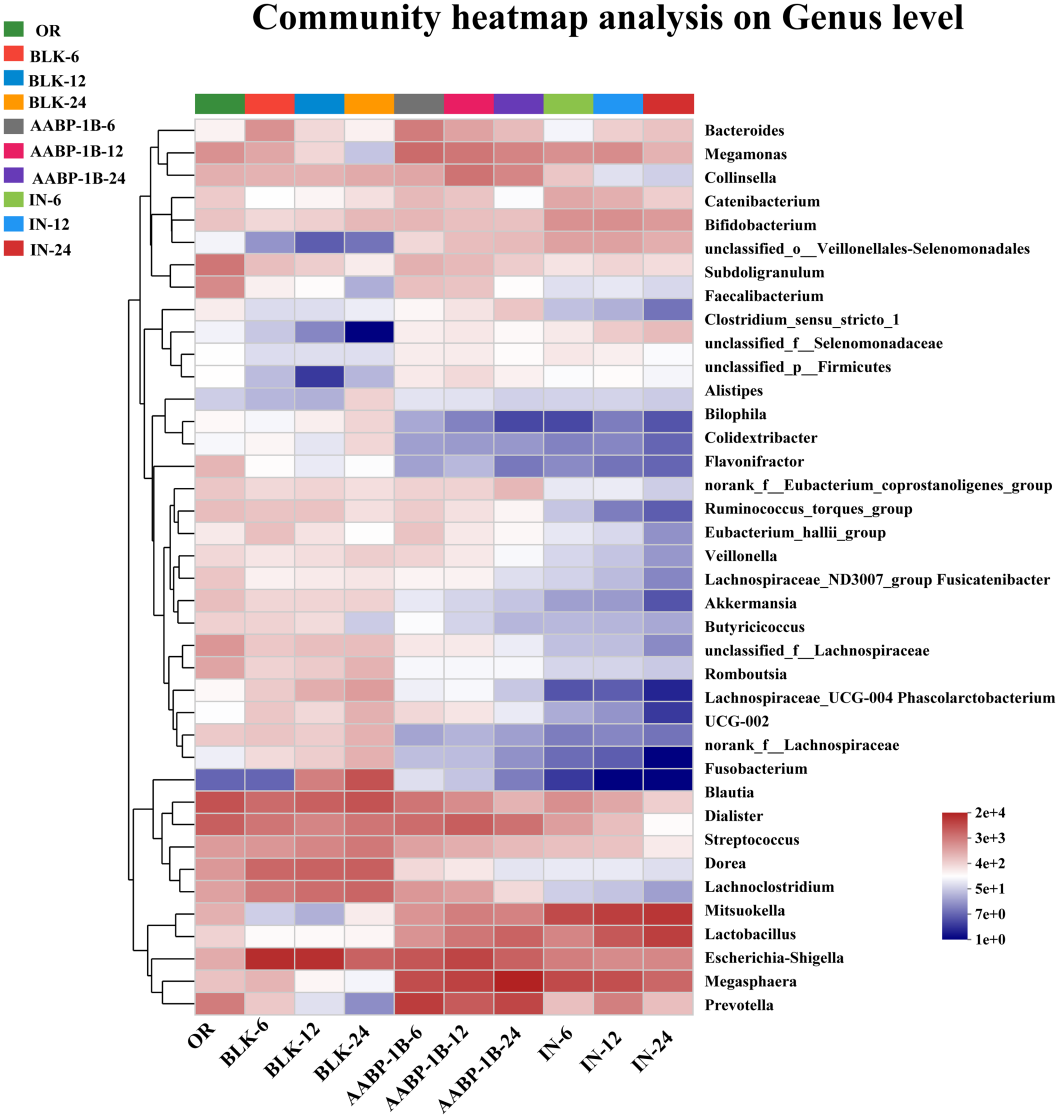
Gut microbiota analysis of AABP-1B during *in vitro* fermentation based on heat map at genus level; OR was blank medium fermentation for 0 h; BLK-6, BLK-12, BLK-24, IN-6, IN-12, IN-24, AABP-1B-6, AABP-1B-12 and AABP-1B-24 were blank medium, Inulin medium, AABP-1B medium fermentation for 6 h, 12 h and 24 h, respectively.

Compared with the BLK group, the AABP-1B group showed higher levels of the genera *Bacteroides*, *Prevotella*, *Lactobacillus*, *Megamonas*, *Megasphaera*, and *Faecalibacterium* (Figure 8). High-fiber diets are associated with high *Prevotella* levels, which may mitigate glucose intolerance induced by *Bacteroides* and enhance glycogen storage in certain demographic groups (54). Thus, the clinical augmentation of *Prevotella* abundance within the intestinal microbiota may aid in the management of blood sugar levels and reduce the likelihood of developing diabetes (55). *Lactobacillus* can improve intestinal barrier integrity and reduce inflammation (56). *Megasphaera* can promote the formation of butyric acid (57, 58). Notably, *Phascolarctobacterium*, a genus within the phylum Firmicutes, was enriched at 24 h. This genus is known to produce propionic acid via the succinate pathway and may contribute to the observed increase in propionic acid levels (59) and is negatively correlated with oral ulcer occurrence (60). These findings align with those of previous studies on SCFAs. Compared with the BLK group, the AABP-1B group showed significantly lower levels of pathogens: the abundances of *Escherichia-Shigella*, *Blautia*, *Lachnoclostridium*, and *Romboutsia* decreased after 24 h of fermentation. Previous studies have linked these detrimental Gram-negative bacteria to intestinal infections, type 2 diabetes, and intestinal complications. *Escherichia-Shigella* has been shown to cause serious diseases by producing various toxins. A strong association has been observed between type 2 diabetes occurrence and *Blautia* abundance (61). *Lachnoclostridium* abundance has been linked to obesity (62). Overall, these findings suggest that AABP-1B may improve intestinal health by altering the composition and diversity of beneficial and pathogenic intestinal bacteria.

**Figure 9:**
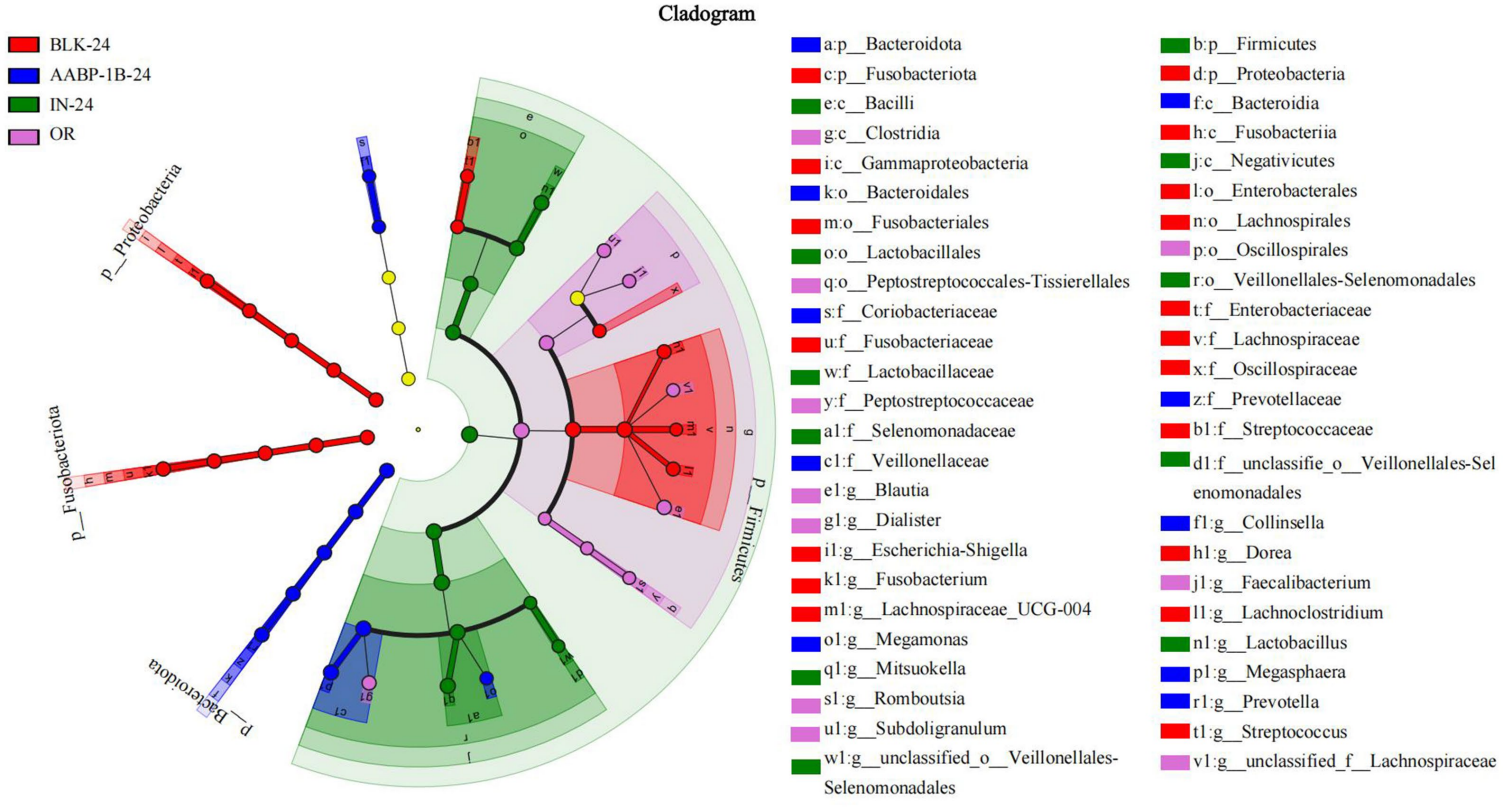
The differences in gut microbes were compared based on LEfSe; LEfSe among Blank 0 (OR), Blank 24 (BLK-24), Inulin 24 (IN-24) and AABP-1B 24 (AABP-1B-24) at OTUs level; Blank 0 was blank medium fermentation for 0 h, Blank 24, Inulin 24, AABP-1B 24 was blank medium, Inulin medium, AABP-1B medium fermentation for 24 h, respectively.

**Figure 10:**
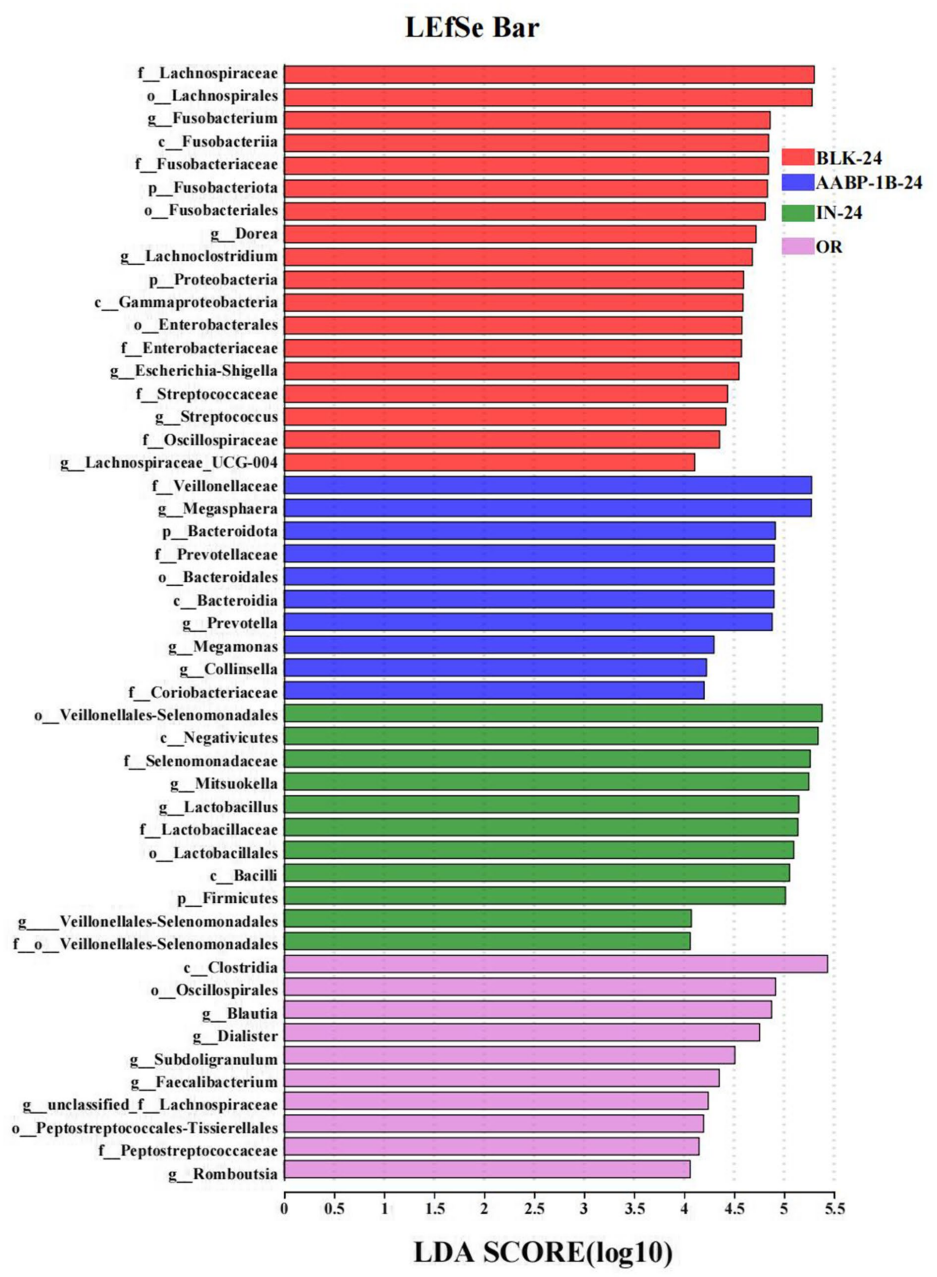
LDA score; LDA among Blank 0 (OR), Blank 24 (BLK-24), Inulin 24 (IN-24) and AABP-1B 24 (AABP-1B-24) at OTUs level; Blank 0 was blank medium fermentation for 0 h, Blank 24, Inulin 24, AABP-1B 24 was blank medium, Inulin medium, AABP-1B medium fermentation for 24 h, respectively.

The LEfSe analysis at the genus level based on LDA scores was investigated to determine the specific gut microbiota affected by Blank 0 (OR), Blank 24 (BLK-24), Inulin 24 (IN-24) and AABP-1B 24 (AABP-1B-24) groups. Different LDA scores were used to represent the significant effects of different species between groups, and the significant differences in species with LDA scores >4 were biomarkers with statistical differences. The results of LEfSe were shown in Figures 9, 10. We identified 49 taxa in the four groups with significant species differences (LDA > 4, Figure 10), including 10 dominant taxa in the AABP-1B group, 11 dominant taxa in the IN group, and 18 dominant taxa in the BLK group. The dominant bacteria in the BLK group were *Lachnospiracea*, *Fusobacteria*, and *Escherichia-Shigella*. The IN group mainly comprised *Selenomonadales*, *Lactobacillaceae*, and *Firmicutes*. The AABP-1B group primarily comprised *Megasphaera*, *Prevotella*, and *Bacteroidota*. Therefore, AABP-1B and inulin affected the composition of the gut microbiota. The beneficial bacteria *Bacteroidota* and *Prevotella* were the dominant strains in the AABP-1B group (Figure 10), indicating that AABP-1B exerts a strong regulatory effect on microbial composition. These findings highlight the potential of AABP-1B for regulating the gut microbiome and promoting the growth of beneficial bacteria, ultimately promoting overall gut health.

## 4 Discussion

In this study, we simulated the *in vitro* environment of the oral, gastric, and small intestinal phases to evaluate the physicochemical and structural motif alterations of *Anemarrhena asphodeloides* Bunge polysaccharide (AABP-1B). These changes included monosaccharide composition, and molecular weight, which collectively influence its prebiotic potential. During the transition from oral to gastrointestinal phases, non-starch polysaccharides (NSPs) undergo sequential structural alterations driven by environmental and enzymatic factors. In the simulated oral digestion stage, salivary amylase is the main enzyme in the mouth, which mainly acts on polysaccharides such as starch and breaks them down into dextrins and oligosaccharides. The main chain of AABP-1 was composed of 4,6)-β-d-Gal*p*-(1, 4)-β-d-Man*p*-(1, 4)-β-d-GalA*p*-(1, 4)-α-l-Rha*p*-(1, T-α-d-Glc*p*-(1, T-α-l-Ara*f*-(1, and 3)-α-l-Ara*f*-(1 and 4)-2-*O*-acetyl-β-d-Manp-(1, and did not contain α-1,4- glucosidic bonds, and thus was resistant to salivary amylase during the oral digestion phase (30). In the gastric phase, although pepsin mainly digests proteins, the strongly acidic environment (pH 1.5–3.5) may induce limited acid-catalyzed hydrolysis of acid-labile glycosidic bonds, including β-1,4 linkages (63). This could explain the slight reduction in molecular weight and the increase in reducing ends observed after gastric digestion. Similarly, polysaccharides from Asian plantain seeds showed decreased molecular weights during gastrointestinal digestion without the release of monosaccharides (64). In the simulated fermentation phase, different kinds of microorganisms carry out complex metabolic activities using AABP-1B as carbon source. In our study, AABP-1B showed the ability to regulate the composition of the gut microbiome, promoting the growth of beneficial microbiomes while inhibiting the proliferation of potentially harmful microbiomes. In addition, the metabolites of AABP-1B are mainly short chain fatty acids such as acetic acid, propionic acid and butyric acid. Acetic acid is predominantly synthesized by *Bacteroides* and *Lactobacillus* species (65, 66). It plays a crucial role in supplying energy to colonic epithelial cells and in strengthening the integrity of the intestinal barrier (67, 68). Propionate acid is linked to the proliferation of microbial taxa such as *Dialister* and *Phascolarctobacterium* and is implicated in the regulation of gluconeogenesis and cholesterol metabolism (69). Propionic acid exerts beneficial effects on diabetes-related metabolic abnormalities through diverse mechanisms, including the enhancement of insulin sensitivity, regulation of glucose and lipid metabolism, attenuation of inflammation, and restoration of intestinal microbiota balance (70–73). For example, propionic acid enhances insulin sensitivity and reduces insulin resistance in peripheral tissues by activating free fatty acid receptors and promoting the secretion of glucagon-like peptide-1 (GLP-1) (74–76). In addition, butyric acid exhibits significant anti-inflammatory properties by modulating the nuclear factor kappa B (TLR4/NF-κB) signaling pathway, thereby reducing the production of pro-inflammatory cytokines (77). In summary, the polysaccharides from *Anemarrhena asphodeloides* Bunge (AABP-1B) can regulate the composition of intestinal flora and the production of short-chain fatty acids. These effects jointly maintain intestinal microecological balance and promote intestinal health, and provide a strong theoretical basis for the development of functional foods and drugs based on polysaccharide, which is worthy of further research, development and utilization.

## 5 Conclusion

This study examined the *in vitro* digestion of AABP-1B and associated changes in the composition of intestinal microbiota during fermentation. Our findings indicated that AABP-1B was only slightly degraded, without yielding free monosaccharides. Its inhibitory effects on α-glucosidase activity remained consistent before and after digestion. After 24 h of fermentation, AABP-1B lowered pH, increased acetic and propionic acid levels, and promoted the growth of beneficial microbes like Prevotella and Megasphaera, suggesting its potential as a prebiotic for intestinal health and functional food development.

## Data Availability

The data presented in the study are deposited in the NCBI Sequence Read Archive (SRA), accession number PRJNA1298559 (https://www.ncbi.nlm.nih.gov/bioproject/PRJNA1298559).

## References

[1] Goodrich JK Waters JL Poole AC Sutter JL Koren O Blekhman R. Human genetics shape the gut microbiome. Cell. (2014) 159:789–99. doi: 10.1016/j.cell.2014.09.053, PMID: 25417156 PMC4255478

[2] Kurilshikov A Medina-Gomez C Bacigalupe R Radjabzadeh D Wang J Demirkan A. Large-scale association analyses identify host factors influencing human gut microbiome composition. Nat Genet. (2021) 53:156–65. doi: 10.1038/s41588-020-00763-1, PMID: 33462485 PMC8515199

[3] Zhang D Liu J Cheng H Wang H Tan Y Feng W. Interactions between polysaccharides and gut microbiota: a metabolomic and microbial review. Food Res Int. (2022) 160:111653. doi: 10.1016/j.foodres.2022.111653, PMID: 36076442

[4] Yang G Wei J Liu P Zhang Q Tian Y Hou G. Role of the gut microbiota in type 2 diabetes and related diseases. Metabolism. (2021) 117:154712. doi: 10.1016/j.metabol.2021.154712 33497712

[5] Zhao L Yu J Liu Y Liu Y Zhao Y Li MY. The major roles of intestinal microbiota and TRAF6/NF-κB signaling pathway in acute intestinal inflammation in mice, and the improvement effect by *Hippophae rhamnoides* polysaccharide. Int J Biol Macromol. (2025) 296:139710. doi: 10.1016/j.ijbiomac.2025.139710, PMID: 39793780

[6] Abdelgani S Khattab A Adams J Abu-Farha M Daniele G Al-Mulla F. Distinct mechanisms responsible for the increase in glucose production and ketone formation caused by Empagliflozin in T2DM patients. Diabetes Care. (2023) 46:978–84. doi: 10.2337/dc22-0885, PMID: 36857415 PMC10154659

[7] Ma Q Li Y Li P Wang M Wang J Tang Z. Research progress in the relationship between type 2 diabetes mellitus and intestinal flora. Biomed Pharmacother (1019). 117:109138. doi: 10.1016/j.biopha.2019.109138 31247468

[8] Yu Z Zhao L Zhao J-L Xu W Guo Z Zhang A-Z. Dietary *Taraxacum mongolicum* polysaccharide ameliorates the growth, immune response, and antioxidant status in association with NF-κB, Nrf2 and TOR in Jian carp (*Cyprinus carpio* var. Jian). Aquaculture. (2021) 547:737522. doi: 10.1016/j.aquaculture.2021.737522

[9] Li M Chen L Zhao Y Sun H Zhao L. Research on the mechanism of HRP relieving IPEC-J2 cells immunological stress based on transcriptome sequencing analysis. Front Nutr. (2022) 9:944390. doi: 10.3389/fnut.2022.944390, PMID: 35911118 PMC9336541

[10] Zhou W Chen G Chen D Ye H Zeng X. The antidiabetic effect and potential mechanisms of natural polysaccharides based on the regulation of gut microbiota. J Funct Foods. (2020) 75:104222. doi: 10.1016/j.jff.2020.104222

[11] Chuanboding Wang N He H Sun X Bi X Li A. Advances in the treatment of type 2 diabetes mellitus by natural plant polysaccharides through regulation of gut microbiota and metabolism: a review. Int J Biol Macromol. (2024) 274:133466. doi: 10.1016/j.ijbiomac.2024.133466 38942411

[12] Floris AL Eloy HL Peter LL Guy ER Chris W Akkermans RP. Alpha-glucosidase inhibitors for patients with type 2 diabetes: results from a Cochrane systematic review and meta-analysis. Diabetes Care. (2005) 28:154–63. doi: 10.2337/diacare.28.1.154 15616251

[13] Yin C Noratto GD Fan X Chen Z Yao F Shi D. The impact of mushroom polysaccharides on gut microbiota and its beneficial effects to host: a review. Carbohydr Polym. (2020) 250:116942. doi: 10.1016/j.carbpol.2020.116942, PMID: 33049854

[14] Liu X Su S Yao J Zhang X Wu Z Jia L. Research advance about plant polysaccharide prebiotics, benefit for probiotics on gut homeostasis modulation. Food Biosci. (2024) 59:103831. doi: 10.1016/j.fbio.2024.103831

[15] Wardman JF Bains RK Rahfeld P Withers SG. Carbohydrate-active enzymes (CAZymes) in the gut microbiome. Nat Rev Microbiol. (2022) 20:542–56. doi: 10.1038/s41579-022-00712-1, PMID: 35347288

[16] Ye K Fu C Ma S Du H Chen S Liu D. Comprehensive assessment of *Hypsizygus marmoreus* polysaccharides through simulated digestion and gut microbiota fermentation in vitro. Food Hydrocoll. (2023) 144:108989. doi: 10.1016/j.foodhyd.2023.108989

[17] Ge Q Hou C-l Rao X-h Zhang A-q Xiao G-m Wang L-y. In vitro fermentation characteristics of polysaccharides from coix seed and its effects on the gut microbiota. Int J Biol Macromol. (2024) 262:129994. doi: 10.1016/j.ijbiomac.2024.129994, PMID: 38325690

[18] Dalile B Van Oudenhove L Vervliet B Verbeke K. The role of short-chain fatty acids in microbiota-gut-brain communication. Nat Rev Gastroenterol Hepatol. (2019) 16:461–78. doi: 10.1038/s41575-019-0157-3, PMID: 31123355

[19] Tan J McKenzie C Potamitis M Thorburn AN Mackay CR Macia L. The role of short-chain fatty acids in health and disease. Advances in immunology (2014). 121:91–119. doi: 10.1016/B978-0-12-800100-4.00003-9 24388214

[20] Patterson E Ryan PM Cryan JF Dinan TG Ross RP Fitzgerald GF. Gut microbiota, obesity and diabetes. Postgrad Med J. (2016) 92:286–300. doi: 10.1136/postgradmedj-2015-133285, PMID: 26912499

[21] Tian B Zhou X Geng Y Hu J Ye B Sun P. Characterization and in vitro digestion of alkali-extracted polysaccharides from *Grifola frondosa* and its impacts on human gut microbiota. Food Biosci. (2024) 60:104499. doi: 10.1016/j.fbio.2024.104499

[22] Ma G Du H Hu Q Yang W Pei F Xiao H. Health benefits of edible mushroom polysaccharides and associated gut microbiota regulation. Crit Rev Food Sci Nutr. (2022) 62:6646–63. doi: 10.1080/10408398.2021.1903385, PMID: 33792430

[23] Zhang Z Lin T Meng Y Hu M Shu L Jiang H. FOS/GOS attenuates high-fat diet induced bone loss via reversing microbiota dysbiosis, high intestinal permeability and systemic inflammation in mice. Metab Clin Exp. (2021) 119:154767. doi: 10.1016/j.metabol.2021.154767, PMID: 33753088

[24] Lan T Tang T Li Y Duan Y Yuan Q Liu W. FTZ polysaccharides ameliorate kidney injury in diabetic mice by regulating gut-kidney axis. Phytomedicine. (2023) 118:154935. doi: 10.1016/j.phymed.2023.154935, PMID: 37364420

[25] Li Q Liu W Zhang H Chen C Liu R Hou H. Α-D-1,3-glucan from Radix *Puerariae thomsonii* improves NAFLD by regulating the intestinal flora and metabolites. Carbohydr Polym. (2023) 299:120197. doi: 10.1016/j.carbpol.2022.120197, PMID: 36876767

[26] Feng X Guo M Li J Shen Z Mo F Tian Y. The structural characterization of a novel Chinese yam polysaccharide and its hypolipidemic activity in HFD-induced obese C57BL/6J mice. Int J Biol Macromol. (2024) 265:130521. doi: 10.1016/j.ijbiomac.2024.130521, PMID: 38553396

[27] Ma Y Xie H Xu N Li M Wang L Ge H. Large yellow tea polysaccharide alleviates HFD-induced intestinal homeostasis dysbiosis via modulating gut barrier integrity, immune responses, and the gut microbiome. J Agric Food Chem. (2024) 72:7230–43. doi: 10.1021/acs.jafc.4c00616, PMID: 38494694

[28] Lan T Wu J Tang B He X Ding X Ren X. Fufang zhenzhu tiaozhi polysaccharides ameliorates high-fat diet-induced non-alcoholic steatohepatitis and intestinal flora disorders in mice. J Funct Foods. (2024) 117:106247. doi: 10.1016/j.jff.2024.106247

[29] Bao Y Shang X Hu G Wang J Liu C Lv Q. *Stevia rebaudiana* root polysaccharide modulates liver metabolism, bile acid, and gut microbiota improving HFD-induced NAFLD: potential roles of ACSL1 and FADS2. Phytomedicine. (2025) 141:156680. doi: 10.1016/j.phymed.2025.156680, PMID: 40220428

[30] Chen J Li L Zhang X Zhang Y Zheng Q Lan M. Structural characteristics and antioxidant and hypoglycemic activities of a heteropolysaccharide from Anemarrhena asphodeloides Bunge. Int J Biol Macromol. (2023) 236:123843. doi: 10.1016/j.ijbiomac.2023.123843, PMID: 36858093

[31] Wu DT Yuan Q Guo H Fu Y Li F Wang SP. Dynamic changes of structural characteristics of snow chrysanthemum polysaccharides during in vitro digestion and fecal fermentation and related impacts on gut microbiota. Food Res Int. (2021) 141:109888. doi: 10.1016/j.foodres.2020.109888, PMID: 33641944

[32] Chen J Lan M Zhang X Jiao W Chen Z Li L. Effects of simulated in vitro digestion on the structural characteristics, inhibitory activity on α-glucosidase, and fermentation Behaviours of a polysaccharide from Anemarrhena asphodeloides Bunge. Nutrients. (2023) 15:1965. doi: 10.3390/nu15081965, PMID: 37111183 PMC10145594

[33] Ankolekar C Pinto M Greene D Shetty K. In vitro bioassay based screening of antihyperglycemia and antihypertensive activities of *Lactobacillus acidophilus* fermented pear juice. Innov Food Sci Emerg Technol. (2012) 13:221–30. doi: 10.1016/j.ifset.2011.10.008

[34] Zhou W Yan Y Mi J Zhang H Lu L Luo Q. Simulated digestion and fermentation in vitro by human gut microbiota of polysaccharides from bee collected pollen of Chinese wolfberry. J Agric Food Chem. (2018) 66:898–907. doi: 10.1021/acs.jafc.7b05546, PMID: 29313353

[35] Luo Q Li X Li H Kong K Li C Fang Z. Effect of in vitro simulated digestion and fecal fermentation on *Boletus auripes* polysaccharide characteristics and intestinal flora. Int J Biol Macromol. (2023) 249:126461. doi: 10.1016/j.ijbiomac.2023.126461, PMID: 37619676

[36] Zhang Y Wang L Qiu Z Yang Y Wang T Inam M. Comprehensive evaluation of Flammulina velutipes residues polysaccharide based on in vitro digestion and human fecal fermentation. Int J Biol Macromol. (2024) 281:136487. doi: 10.1016/j.ijbiomac.2024.136487, PMID: 39414219

[37] Ma GX Kimatu BM Zhao LY Yang WJ Pei F Hu QH. In vivo fermentation of a *Pleurotus eryngii* polysaccharide and its effects on fecal microbiota composition and immune response. Food Funct. (2017) 8:1810–21. doi: 10.1039/c7fo00341b, PMID: 28513745

[38] Chen M Chen X Guo Y Liu N Wang K Gong P. Effect of in vitro digestion and fermentation of kiwifruit pomace polysaccharides on structural characteristics and human gut microbiota. Int J Biol Macromol. (2023) 253:127141. doi: 10.1016/j.ijbiomac.2023.127141, PMID: 37776924

[39] Zhao T Wang C Liu Y Li B Shao M Zhao W. The role of polysaccharides in immune regulation through gut microbiota: mechanisms and implications. Front Immunol. (2025) 16:1555414. doi: 10.3389/fimmu.2025.1555414 40230839 PMC11994737

[40] Kim CH. Complex regulatory effects of gut microbial short-chain fatty acids on immune tolerance and autoimmunity. Cell Mol Immunol. (2023) 20:341–50. doi: 10.1038/s41423-023-00987-1, PMID: 36854801 PMC10066346

[41] Basen M Kurrer SE. A close look at pentose metabolism of gut bacteria. FEBS J. (2021) 288:1804–8. doi: 10.1111/febs.15575, PMID: 33063458

[42] Portincasa P Bonfrate L Vacca M De Angelis M Farella I Lanza E. Gut microbiota and short chain fatty acids: implications in glucose homeostasis. Int J Mol Sci. (2022) 23:1105. doi: 10.3390/ijms23031105, PMID: 35163038 PMC8835596

[43] Rauf A Khalil AA Rahman U-u Khalid A Naz S Shariati MA. Recent advances in the therapeutic application of short-chain fatty acids (SCFAs): an updated review. Crit Rev Food Sci Nutr. (2022) 62:6034–54. doi: 10.1080/10408398.2021.1895064, PMID: 33703960

[44] Han R Pang D Wen L You L Huang R Kulikouskaya V. In vitro digestibility and prebiotic activities of a sulfated polysaccharide from *Gracilaria lemaneiformis*. J Funct Foods. (2020) 64:103652. doi: 10.1016/j.jff.2019.103652

[45] Al-Lahham SAH Peppelenbosch MP Roelofsen H Vonk RJ Venema K. Biological effects of propionic acid in humans; metabolism, potential applications and underlying mechanisms. Biochim Biophys Acta. (2010) 1801:1175–83. doi: 10.1016/j.bbalip.2010.07.007 20691280

[46] Chen C Su Y Li S Man C Jiang Y Qu B. Advances in oligosaccharides and polysaccharides with different structures as wall materials for probiotics delivery: a review. Int J Biol Macromol. (2024) 277:134468. doi: 10.1016/j.ijbiomac.2024.134468, PMID: 39217037

[47] Dong J Wang W Zheng G Wu N Xie J Xiong S. In vitro digestion and fermentation behaviors of polysaccharides from *Choerospondias axillaris* fruit and its effect on human gut microbiota. Curr Res Food Sci. (2024) 8:100760. doi: 10.1016/j.crfs.2024.100760, PMID: 38764977 PMC11098719

[48] Shin N-R Whon TW Bae J-W. Proteobacteria: microbial signature of dysbiosis in gut microbiota. Trends Biotechnol. (2015) 33:496–503. doi: 10.1016/j.tibtech.2015.06.011, PMID: 26210164

[49] Hou K Wu Z-X Chen X-Y Wang J-Q Zhang D Xiao C. Microbiota in health and diseases. Signal Transduct Target Ther. (2022) 7:135. doi: 10.1038/s41392-022-00974-4, PMID: 35461318 PMC9034083

[50] Aljahdali NH Sanad YM Han J Foley SL. Current knowledge and perspectives of potential impacts of *Salmonella enterica* on the profile of the gut microbiota. BMC Microbiol. (2020) 20:353. doi: 10.1186/s12866-020-02008-x, PMID: 33203384 PMC7673091

[51] Lapébie P Lombard V Drula E Terrapon N Henrissat B. Bacteroidetes use thousands of enzyme combinations to break down glycans. Nat Commun. (2019) 10:2043. doi: 10.1038/s41467-019-10068-5, PMID: 31053724 PMC6499787

[52] Muheyati D Han J Lv M Jielili M Jing Z Zaibibuli K. Composition of gut microbiota in obese and normal-weight Uygur adults and its association with adipocyte-related factors. Sci Rep. (2024) 14:24649. doi: 10.1038/s41598-024-76351-8, PMID: 39428421 PMC11491455

[53] Karačić A Renko I Krznarić Ž Klobučar S Liberati Pršo AM. The association between the Firmicutes/Bacteroidetes ratio and body mass among European population with the highest proportion of adults with obesity: an observational follow-up study from Croatia. Biomedicines. (2024) 12:2263. doi: 10.3390/biomedicines12102263, PMID: 39457576 PMC11505267

[54] Kovatcheva-Datchary P Nilsson A Akrami R Lee YS De Vadder F Arora T. Dietary Fiber-induced improvement in glucose metabolism is associated with increased abundance of Prevotella. Cell Metab. (2015) 22:971–82. doi: 10.1016/j.cmet.2015.10.001, PMID: 26552345

[55] Ortega-Santos CP Whisner CM. The key to successful weight loss on a high-fiber diet may be in gut microbiome *Prevotella* abundance. J Nutr. (2019) 149:2083–4. doi: 10.1093/jn/nxz248, PMID: 31584088

[56] Zhang H Pertiwi H Hou Y Majdeddin M Michiels J. Protective effects of *Lactobacillus* on heat stress-induced intestinal injury in finisher broilers by regulating gut microbiota and stimulating epithelial development. Sci Total Environ. (2024) 918:170410. doi: 10.1016/j.scitotenv.2024.170410, PMID: 38280596

[57] Liu J Lu X Fang F Wu K Wu J Gao J. Comparison of the in-vitro effect of five prebiotics with different structure on gut microbiome and metabolome. Food Biosci. (2024):103810. doi: 10.1016/j.fbio.2024.103810

[58] Hashizume K Tsukahara T Yamada K Koyama H Ushida K. *Megasphaera elsdenii* JCM1772T normalizes Hyperlactate production in the large intestine of Fructooligosaccharide-fed rats by stimulating butyrate production. J Nutr. (2003) 133:3187–90. doi: 10.1093/jn/133.10.3187, PMID: 14519808

[59] Ogata Y Suda W Ikeyama N Hattori M Ohkuma M Sakamoto M. Complete genome sequence of *Phascolarctobacterium faecium* JCM 30894, a succinate-utilizing bacterium isolated from human feces. Microbiol Resour Announc. (2019) 8:e01487-18. doi: 10.1128/mra.01487-18, PMID: 30687834 PMC6346166

[60] Jin B Wang P Liu P Wang Y Guo Y Wang C. Genetic connectivity of gut microbiota and Oral ulcers: a Mendelian randomization study. Int Dent J. (2024) 74:696–704. doi: 10.1016/j.identj.2024.02.007, PMID: 38458846 PMC11287153

[61] Gurung M Li Z You H Rodrigues R Jump DB Morgun A. Role of gut microbiota in type 2 diabetes pathophysiology. EBioMedicine. (2020) 51:102590. doi: 10.1016/j.ebiom.2019.11.051, PMID: 31901868 PMC6948163

[62] Zhang X-Y Chen J Yi K Peng L Xie J Gou X. Phlorizin ameliorates obesity-associated endotoxemia and insulin resistance in high-fat diet-fed mice by targeting the gut microbiota and intestinal barrier integrity. Gut Microbes. (2020) 12:1842990–18. doi: 10.1080/19490976.2020.1842990, PMID: 33222603 PMC7714487

[63] Nguyen HSH Heinonen J Laatikainen M Sainio T. Evolution of the molar mass distribution of oat β-glucan during acid catalyzed hydrolysis in aqueous solution. Chem Eng J. (2020) 382:122863. doi: 10.1016/j.cej.2019.122863

[64] Hu J-L Nie S-P Min F-F Xie M-Y. Artificial simulated saliva, gastric and intestinal digestion of polysaccharide from the seeds of *Plantago asiatica* L. Carbohydr Polym. (2013) 92:1143–50. doi: 10.1016/j.carbpol.2012.10.072, PMID: 23399139

[65] Chen C Niu M Pan J Du N Liu S Li H. Bacteroides, butyric acid and t10,c12-CLA changes in colorectal adenomatous polyp patients. Gut Pathog. (2021) 13:1. doi: 10.1186/s13099-020-00395-0, PMID: 33436066 PMC7805033

[66] Nie Q Sun Y Li M Zuo S Chen C Lin Q. Targeted modification of gut microbiota and related metabolites via dietary fiber. Carbohydr Polym. (2023) 316:120986. doi: 10.1016/j.carbpol.2023.120986, PMID: 37321707

[67] Valdes DS So D Gill PA Kellow NJ. Effect of dietary acetic acid supplementation on plasma glucose, lipid profiles, and body mass index in human adults: a systematic review and meta-analysis. J Acad Nutr Diet. (2021) 121:895–914. doi: 10.1016/j.jand.2020.12.002, PMID: 33436350

[68] Kimura I Ozawa K Inoue D Imamura T Kimura K Maeda T. The gut microbiota suppresses insulin-mediated fat accumulation via the short-chain fatty acid receptor GPR43. Nat Commun. (2013) 4:1829. doi: 10.1038/ncomms2852, PMID: 23652017 PMC3674247

[69] Louis P Hold GL Flint HJ. The gut microbiota, bacterial metabolites and colorectal cancer. Nat Rev Microbiol. (2014) 12:661–72. doi: 10.1038/nrmicro3344, PMID: 25198138

[70] Koh A Molinaro A Ståhlman M Khan MT Schmidt C Mannerås-Holm L. Microbially produced imidazole propionate impairs insulin signaling through mTORC1. Cell. (2018) 175:947–961.e17. doi: 10.1016/j.cell.2018.09.055, PMID: 30401435

[71] Shu T Song X Zhang S Zhou X Zhang Z Wang P. Screen of propionate-producing probiotic strains in attenuating murine colitis by modulating immune response and restoring the intestinal barrier. J Funct Foods. (2025) 125:106660. doi: 10.1016/j.jff.2025.106660

[72] Wu Q Dong J Bai X Jiang Y Li J Fan S. Propionate ameliorates diabetes-induced neurological dysfunction through regulating the PI3K/Akt/eNOS signaling pathway. Eur J Pharmacol. (2022) 925:174974. doi: 10.1016/j.ejphar.2022.174974, PMID: 35490725

[73] Yang F Xia N Guo S Zhang J Liao Y Tang T. Propionate alleviates abdominal aortic aneurysm by modulating colonic regulatory T-cell expansion and recirculation. JACC Basic Transl Sci. (2022) 7:934–47. doi: 10.1016/j.jacbts.2022.05.001, PMID: 36317128 PMC9617133

[74] Wang Q-Y Zhang W Zhao Y Chen H-L Liu Q Wang Z-H. Colonic L-cell impairment in aged subjects with type 2 diabetes leads to diminished GLP-1 production. Diabetes Metab Syndr Clin Res Rev. (2023) 17:102907. doi: 10.1016/j.dsx.2023.102907, PMID: 37980723

[75] Xia T He W Luo Z Wang K Tan X. *Achyranthes bidentata* polysaccharide ameliorates type 2 diabetes mellitus by gut microbiota-derived short-chain fatty acids-induced activation of the GLP-1/GLP-1R/cAMP/PKA/CREB/INS pathway. Int J Biol Macromol. (2024) 270:132256. doi: 10.1016/j.ijbiomac.2024.132256, PMID: 38729481

[76] Yadav H Lee J-H Lloyd J Walter P Rane SG. Beneficial metabolic effects of a probiotic via butyrate-induced GLP-1 hormone secretion*. J Biol Chem. (2013) 288:25088–97. doi: 10.1074/jbc.M113.452516, PMID: 23836895 PMC3757173

[77] Zhang R Yuan S Ye J Wang X Zhang X Shen J. Polysaccharide from flammuliana velutipes improves colitis via regulation of colonic microbial dysbiosis and inflammatory responses. Int J Biol Macromol. (2020) 149:1252–61. doi: 10.1016/j.ijbiomac.2020.02.044, PMID: 32035958

