# Supplementary material for: Effects of in vitro simulated digestion on the α-glucosidase inhibitory activity, structure, and prebiotic activity of a polysaccharide from Anemarrhena asphodeloides Bunge
Source: Front Nutr. 2025 Aug 8;12:1603237. doi: 10.3389/fnut.2025.1603237 (PMC12370768; doi:10.3389/fnut.2025.1603237)
Supplement: Supplementary file 1 [file Table_1.docx]

Appendix Table 1 Changes in Mw and CR of polysaccharides during simulated in vitro digestion

|  | Time（h） | Molecular weight (KDa) | Reducing sugar (mg/mL) |
| --- | --- | --- | --- |
| Salivary digestion | 0 | 105.01±0.57^a^ | 0.502±0.012^b^ |
|  | 0.5 | 105.8±0.43^a^ | 0.511±0.021^b^ |
| Gastric digestion | 2 | 98.2±0.24^b^ | 0.732±0.011^a^ |
|  | 4 | 98.5±0.87^b^ | 0.753±0.027^a^ |
|  | 6 | 98.7±0.49^b^ | 0.748±0.014^a^ |
| Intestinal digestion | 2 | 97.6±0.77^b^ | 0.767±0.009^b^ |
|  | 4 | 97.3±0.59^b^ | 0.755±0.018^b^ |

*Different letters within the same column indicate significant differences (*p* < 0.05).

Appendix Table 2 Changes in Short-Chain Fatty Acids During Fermentation

| Sample | Time（h） | Short-Chain Fatty Acid Content (mmol/L) | | | | | |
| --- | --- | --- | --- | --- | --- | --- | --- |
|  |  | Acetic acid | Propionic acid | n-butyric acid | Isobutyric acid | n-pentanoic acid | total SCFAs |
| BLK | 0 | 0.12±0.36^h^ | 0.34±0.11^g^ | 0.23±0.05^g^ | 0.08±0.03^h^ | 0.22±0.04^d^ | 1.18±0.24^g^ |
|  | 6 | 1.53±0.06^f^ | 2.02±0.12^f^ | 0.83±0.12^e^ | 0.12±0.04^g^ | 0.47±0.08^c^ | 5.15±0.44^f^ |
|  | 12 | 4.33±0.12^e^ | 7.43±0.21^d^ | 2.85±0.10^d^ | 0.24±0.05^e^ | 1.24±0.22^b^ | 16.34±0.63^d^ |
|  | 24 | 5.25±0.14^d^ | 8.59±0.36^c^ | 3.30±0.43^c^ | 0.28±0.10^d^ | 1.84±0.19^a^ | 19.48±0.30^c^ |
| AABP-1B | 0 | 0.18±0.05^g^ | 0.36±0.12^g^ | 0.28±0.07^f^ | 0.16±0.05^f^ | 0.07±0.02^g^ | 1.14±0.27^h^ |
|  | 6 | 6.82±0.17^c^ | 3.27±0.24^e^ | 3.06±0.17^c^ | 0.34±0.09^c^ | 0.03±0.01^h^ | 13.60±0.37^e^ |
|  | 12 | 18.5±0.49^b^ | 9.59±0.34^b^ | 5.64±0.32^a^ | 0.41±0.09^a^ | 0.12±0.03^f^ | 34.36±1.27^b^ |
|  | 24 | 19.37±0.80^a^ | 11.59±0.59^a^ | 5.53±0.32^b^ | 0.37±0.08^b^ | 0.17±0.06^e^ | 37.15±0.27^a^ |
